# On Anacahuite Wood

**Published:** 1861-04

**Authors:** Jno. M. Maisch


### *On Anacahuite Wood.* By Jno. M. Maisch.

Some time in August or September last, the New York "Criminalzeitung" published a correspondence from Berlin, Germany, which contained a statement to the effect that the Prussian consul at Tampico, Mexico, had notified his government of a wood, which is called there anacahuite, and is extensively employed with the most beneficial results in tubercular consumption. This report was considered by the government of Prussia of sufficient importance to determine on testing its efficacy in the Charité, the celebrated hospital in Berlin. The wood, it is asserted, is rare in Mexico, and the agents of Prussia have seized upon it to such an extent, that it is now difficult for others to obtain.

A subsequent correspondence of the above mentioned paper, dated Berlin, Oct. 5th, 1860, stated that the experiments with the anacahuite wood had, as yet, not led to a satisfactory result, but that they were to be continued, because it was hardly to be expected that the consul should have made such positive assertions without being satisfied of their correctness; it was added that guaiac wood, had, by some druggists in Germany, been fradulently sold instead of anacahuite.

The "Criminalzeitung" of February 1st, publishes a paper by Dr. Krog, of New York, formerly of Berlin, which appears to be of so much interest as warrant its translation for the American Journal of Pharmacy.

"During the summer of the past year, when the above wood was first brought to Europe, the writer has had the opportunity to observe its effects in Berlin Charite, and to satisfy himself of the unusually favorable results. The first experiments did not prove to be so effectual as was expected, and it was supposed that a part, at least, of the alleged excellent success in Mexico must be ascribed to its climate; but subsequently it became evident that this difference was to be accounted for by the mode of preparing the wood. A simple infusion is not sufficient to extract the active principles; even heating to boiling will not entirely accomplish the object, which is attained only by continuing the boiling for a quarter or half an hour. Such a decoction has proved very efficacious; it produced the complete resorption of the tubercles in the stage of consumption, and afforded great relief of their distressing condition to those farther advanced. These facts suffice to make it desirable that this remedy might be extensively employed here for the benefit of the numerous patients of this class."

Such information, coming from a medical man who had witnessed the experiments, is entitled to consideration, and in view of the importance of the subject, I asked for further information, and received the following letter, which speaks for itself:

New York, Feb. 5th, 1861.

Dear Sir:—In reply to your favor, I hasten to inform you that the anacahuite wood certainly merits the attention which it has lately received. My experience chiefly extends to its medicinal effects. At present I am unable to give the name of the mother-plant; to judge, however, from some pieces of genuine wood now before me, I am inclined to feel justified in placing it in the natural order of Papilionaceæ.

It is believed in Berlin that gratifying results have been obtained with this remedy in the first stages of phthisis pulmonalis. It is given in the form of decoction, namely: ʒ vi. to ℥ i. of the wood to 12 to 14 oz. of water, boiled down to ℥ v., and this is taken 2 to 4 times daily, according to circumstances, combined with other remedies. It is requisite to continue the use of this remedy for several months, and to observe a diet in accordance with the nature of the disease.

Two weeks ago, I wrote to Berlin for the purpose of learning the latest observation with this wood, and I am ready with pleasure to communicate to you the results on their arrival. Yours, &c.,

KROG, M. D., 337 Tenth St.

It is proper, however, to state that Professor Bock, of Leipzig, opposes the use of anacahuite, insisting that in pulmonary consumption relief can *only* be found in the strictest regimen, by partaking of suitable food, breathing a pure and warm atmosphere, using moderate exercise, and attending scrupulously to a mental, intellectual, corporeal, and sexual rest.—*American Journal of Pharmacy.*

